# New Trends of Personalized Medicine in the Management of Abdominal Aortic Aneurysm: A Review

**DOI:** 10.3390/jpm14121148

**Published:** 2024-12-10

**Authors:** Yaman Alsabbagh, Young Erben, Jonathan Vandenberg, Houssam Farres

**Affiliations:** Division of Vascular and Endovascular Surgery, Mayo Clinic, Jacksonville, FL 32224, USA; alsabbagh.yaman@mayo.edu (Y.A.); erben.young@mayo.edu (Y.E.); vandenberg.jonathan@mayo.edu (J.V.)

**Keywords:** biomechanics, stem cell, calcium score, genomics, artificial intelligence, endovascular

## Abstract

Abdominal aortic aneurysm (AAA) is a significant vascular condition characterized by the dilation of the abdominal aorta, presenting a substantial risk of rupture and associated high mortality rates. Current management strategies primarily rely on aneurysm diameter and growth rates to predict rupture risk and determine the timing of surgical intervention. However, this approach has limitations, as ruptures can occur in smaller AAAs below surgical thresholds, and many large AAAs remain stable without intervention. This review highlights the need for more precise and individualized assessment tools that integrate biomechanical parameters such as wall stress, wall strength, and hemodynamic factors. Advancements in imaging modalities like ultrasound elastography, computed tomography (CT) angiography, and magnetic resonance imaging (MRI), combined with artificial intelligence, offer enhanced capabilities to assess biomechanical indices and predict rupture risk more accurately. Incorporating these technologies can lead to personalized medicine approaches, improving decision-making regarding the timing of interventions. Additionally, emerging treatments focusing on targeted delivery of therapeutics to weakened areas of the aortic wall, such as nanoparticle-based drug delivery, stem cell therapy, and gene editing techniques like CRISPR-Cas9, show promise in strengthening the aortic wall and halting aneurysm progression. By validating advanced screening modalities and developing targeted treatments, the future management of AAA aims to reduce unnecessary surgeries, prevent ruptures, and significantly improve patient outcomes.

## 1. Introduction

Abdominal aortic aneurysm (AAA) is a significant vascular condition characterized by dilation of the abdominal aorta exceeding 50% of its normal diameter [1,2]. As the most common site for aneurysm formation, the abdominal aorta saw a global incidence of approximately 35.12 million AAA cases in 2019 [3,4]. Medicare data from 2003 to 2018 highlight that of the 32,760 patients treated for AAA, 28,281 underwent endovascular repair, while 4479 received open repair [5]. Despite advancements in diagnostic and therapeutic approaches, AAA rupture remains life-threatening, with a U.S. incidence rate of 7.29 per 100,000 individuals, a 1-year rupture risk of 9.4%, and an associated mortality rate close to 80% [6,7,8]. This high mortality rate following rupture persists even with the use of proposed therapeutic algorithms aimed at improving outcomes [9].

AAA is notably more prevalent than thoracic or thoracoabdominal aneurysms, occurring five times as often [10]. The infrarenal segment of the abdominal aorta is particularly prone to aneurysmal dilation due to increased peripheral resistance and oscillatory wall shear stress [10,11,12]. This segment’s vulnerability is attributed to its unique hemodynamic environment and structural properties [10].

The pathogenesis of AAA is complex, involving an interplay of genetic predispositions, inflammatory processes, and biomechanical factors that ultimately compromise aortic wall integrity. Risk factors for AAA formation include advanced age, male gender, smoking, hypertension, pulmonary disease, connective tissue disease, alpha-1-antitrypsin deficiency, and atherosclerosis, with smoking identified as a particularly significant modifiable risk factor [4,13,14,15,16].

Current guidelines for AAA management primarily rely on morphometric assessments, focusing on aneurysm diameter and growth rates [17,18]. The Society for Vascular Surgery recommends surgical repair for AAAs ≥5.5 cm in men or ≥5.0 cm in women or for aneurysms exhibiting rapid growth—defined as an increase of 0.5 cm in 6 months or 1 cm in 1 year [18,19]. These guidelines are based on studies that have established a correlation between aneurysm size, growth rates, and rupture risk. However, relying solely on diameter and growth rate has limitations. Ruptures can occur in small AAAs below the surgical threshold. A systematic review reported rupture rates of 1.61 per 100 person-years for small AAAs under the intervention threshold [20]. Laine et al. found that 5.6% of men and 11.5% of women with ruptured AAA had diameters below the recommended thresholds for intervention according to European guidelines [21]. Conversely, many patients present with large AAAs exceeding these thresholds without experiencing rupture [22,23]. This variability suggests that aneurysm diameter alone is insufficient to predict rupture risk. In practice, up to 40% of AAAs are treated before reaching the recommended size for repair [24]. This preemptive approach may lead to unnecessary surgical interventions, exposing patients to procedural risks without justifiable benefits [25]. Scott et al. reported that among patients with AAA >5.5 cm in diameter who did not undergo surgical repair, 64% remained free from rupture or surgery for acute symptoms over five years [26]. This variability in AAA progression and rupture risk underscores the need for more precise and individualized assessment tools.

The limitations of current management strategies highlight the need for personalized medicine approaches in vascular health. Personalized medicine tailors treatment plans based on individual patient characteristics, moving beyond the “one-size-fits-all” approach [27]. In the context of AAA, individualized assessments incorporating biomechanical parameters, tissue properties, and patient-specific anatomy could improve rupture risk predictions [28]. Biomechanical modeling, including finite element analysis (FEA) and wall stress assessments, offers a means to evaluate the mechanical forces acting on the aneurysm wall. By integrating patient-specific data—such as aneurysm geometry, wall thickness, fluid dynamics, and calcification patterns—clinicians can make more informed decisions about when to intervene surgically [29]. This approach aims to reduce unnecessary surgeries and identify high-risk patients who may benefit from earlier intervention. Personalized medicine in AAA management holds the potential to improve patient outcomes, optimize resource utilization, and advance our understanding of aneurysm pathophysiology [30].

This review aims to provide a summary of contemporary personalized medicine approaches in the management of AAA, highlighting advancements in risk prediction through the integration of biomechanical, imaging, and biomarker data, as well as the use of artificial intelligence (AI) to predict AAA behavior and assessing rupture risk.

## 2. Biomechanics

### 2.1. Wall Mechanics

Predicting the rupture risk of an AAA requires a thorough understanding of the balance between wall strain and wall strength. Wall strain refers to the deformation of the aneurysm wall due to internal blood pressure, causing it to stretch, whereas wall strength, refers to the wall’s ability to resist this deformation [2]. A high strain-to-strength ratio indicates a greater risk of rupture. Therefore, an aneurysm is more prone to rupture when it experiences elevated strain or when the wall’s structural integrity is weakened [31,32,33].

The concept of using wall stress as a biomechanical marker for assessing AAAs was introduced and further developed in 2002 by Fillinger et al. They advanced this approach by incorporating data collected from ruptured AAAs to simulate stress distribution [34,35]. Biomechanics were assessed using a computer-based modeling technique to simulate the stress distribution in a theoretical model of AAA [34,36]. Several biomechanical indices have since been established as key indicators of rupture risk. The peak wall stress (PWS), which measures the maximum mechanical stress experienced by the aneurysm wall, is a highly predictive marker for rupture [37,38]. Research has shown that PWS often correlates more directly with rupture than aneurysm diameter, with higher PWS values indicating greater rupture risk [39]. The rupture potential index (RPI) combines wall stress and wall strength into a single metric, allowing clinicians to assess rupture risk by comparing areas of high stress with weakened tissue [10,38,40]. When local stress exceeds the localized strength of the aneurysm wall, the risk of rupture rises significantly [41]. The finite element analysis rupture index (FEARI) uses patient-specific data to model stress distributions in the aneurysm through finite element analysis (FEA) [42]. FEARI accounts for the aneurysm’s unique geometry and material properties, simulating how the wall will behave under physiological pressures and identifying rupture-prone regions [28].

The structural characteristics of the aneurysm wall are major factors influencing rupture risk [43,44]. The geometry of the aneurysm, particularly the asymmetry and tortuosity, significantly impacts how mechanical forces are distributed across the aorta [45]. For instance, asymmetry can cause uneven pressure, concentrating stress in particular areas, increasing susceptibility to rupture [46]. Similarly, a tortuous aneurysm with increased twisting or curvature disrupts blood flow, creating uneven force distribution and further elevating rupture risk [47]. These geometric features are directly linked to aneurysm evolution and rupture potential over time. Wang et al. reported that increased neck angulation is associated with higher peak wall stress and wall shear stress. They observed stress concentrations at the aneurysm’s maximum diameter and the proximal neck [48].

**Figure 1:**
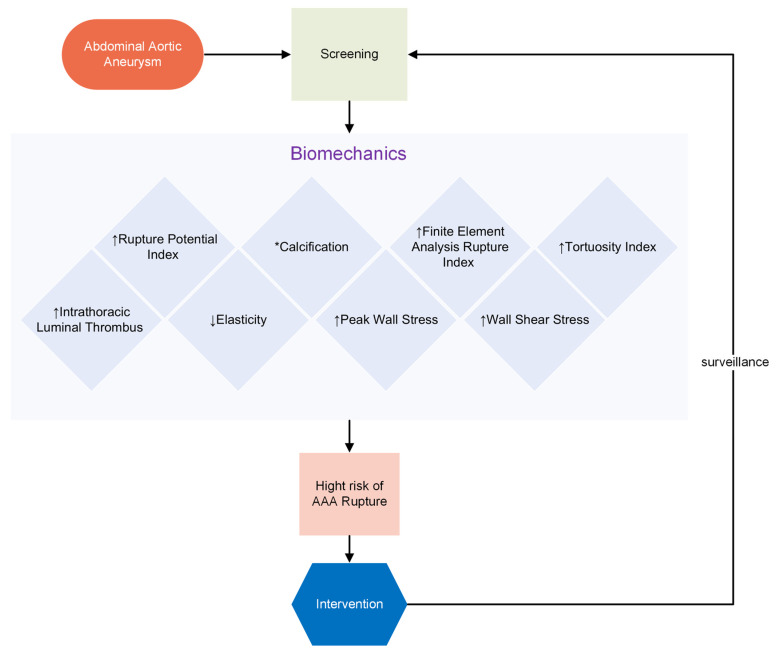
This diagram illustrates the pathway of AAA screening through biomechanics, highlighting the association of various indices with the risk of AAA rupture. An upward arrow (↑) indicates a higher value, while a downward arrow (↓) indicates a lower value. * Literature reports mixed associations. Calcification can stiffen the wall, potentially reducing risk, but areas without calcification may be weaker and more prone to rupture.

Another important element is the elasticity of the aneurysm wall, which can be assessed through elastography. Elastography quantifies the degree to which the aneurysm wall deforms under stress [49]. In healthy tissue, elasticity allows the arterial wall to stretch and recoil, accommodating to internal pressure. However, in aneurysmal tissue, the degradation of structural proteins like elastin and collagen reduces elasticity, leading to stiffness, which hinders stress absorption and increases rupture risk [50]. By quantifying elasticity, elastography provides insight into the aneurysm wall’s strength and ability to withstand mechanical forces [51]. Dong et al. utilized magnetic resonance elastography (MRE) to measure stiffness and found no correlation between AAA diameter and stiffness. However, they noted that aortic rupture was linked to lower AAA stiffness and a decreased stiffness ratio (Figure 1) [52].

### 2.2. Fluid Dynamics

Beyond the structural properties of the wall, the behavior of fluid dynamics within the aneurysm plays a vital role in determining rupture risk. Wall shear stress (WSS) is the frictional force exerted by the movement of blood against the aortic wall. In healthy vessels, WSS helps regulate endothelial function and maintain the integrity of the vessel wall [53]. However, in an aneurysm, altered blood flow leads to areas of low WSS, which promotes the development of intraluminal thrombus (ILT)—a blood clot that forms within the aneurysm sac [53]. ILT can have dual effects: it may reduce wall stress by acting as a mechanical cushion, but it also contributes to the weakening of the wall by inducing inflammation and hypoxia, thereby increasing rupture risk [54,55]. Koncar at al. found in their multivariable analysis that both relative ILT (OR = 1.039) and total aneurysm volume (OR = 1.006) were significant predictors of AAA rupture and PWS [55]. Hemodynamic risks arise from how blood flow patterns inside the aneurysm influence mechanical stress on the wall [56]. When blood flow becomes turbulent due to aneurysm geometry or the presence of ILT, it can create abnormal pressure zones that further increase the wall’s strain [57]. These turbulent flow patterns, coupled with elevated blood pressure or regions of blood stasis, can accelerate aneurysm growth, increasing the likelihood of rupture [58]. Aalbregt et al. utilized 4D flow magnetic resonance imaging (MRI) to assess hemodynamics within AAAs and found that WSS positively correlates with flow velocity and inversely correlates with luminal diameter [59]. The tortuosity index, which quantifies the degree of twisting in the aneurysm, can be a helpful indicator of regions experiencing disturbed flow and heightened stress (Figure 1). Assessing the biomechanics of AAA is challenging because it requires detailed, patient-specific data on aneurysm geometry and wall material properties, which are difficult to obtain non-invasively (Table 1). Due to ethical and practical constraints, validation studies are limited, often relying on retrospective comparisons between ruptured and unruptured cases [29].

### 2.3. Imaging Modalities

**Figure 2:**
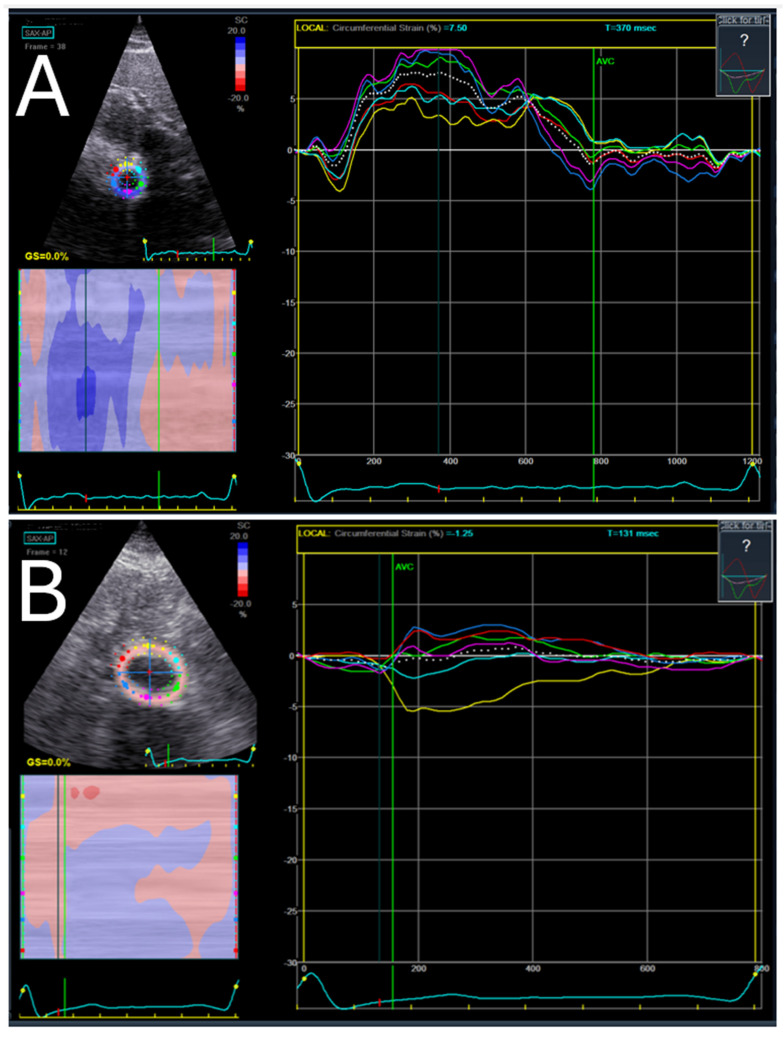
Comparison of strain data between non-aneurysmal (**A**) and aneurysmal (**B**) aorta. The figure illustrates a marked difference in strain curve homogeneity; the non-aneurysmal aorta (**A**) exhibits a more uniform strain distribution, whereas the aneurysmal aorta (**A**) displays heterogeneous strain curves. This increased heterogeneity suggests regions of higher stress.

To assess AAA rupture risk, various imaging modalities are utilized to measure key biomechanical and hemodynamic parameters. Computed tomography (CT) angiography is the most widely used imaging technique for evaluating aneurysm geometry, including symmetry, tortuosity, and wall thickness [60,61]. It also provides the data required for FEA to estimate PWS and RPI, allowing for detailed modeling of stress distributions [62,63]. MRI, particularly phase-contrast MRI, is used to assess blood flow dynamics and can be employed to calculate WSS. Additionally, MRI can be useful for assessing the elasticity of the aortic wall, especially with dynamic imaging techniques like elastography [64,65]. Ultrasound, including Doppler ultrasound, is often used for real-time evaluation of blood flow and WSS, particularly in patients undergoing routine monitoring [66,67,68,69,70,71,72]. Elastography using speckle-tracking imagining (STI) using ultrasound can also be used to evaluate the mechanical properties of the aneurysm wall, including stiffness and elasticity, helping to determine the wall’s capacity to withstand stress [73,74]. To perform STI and measure strain in the abdominal aorta, the process involves acquiring short-axis views of the aneurysm and non-aneurysmal aortic segments using a 2D ultrasound machine. The images are then analyzed using speckle-tracking software (such as GE EchoPac software (Version 201)), where a region of interest (ROI) is manually defined along the aortic wall. The software tracks the movement of speckles (small natural features) within the ROI throughout the cardiac cycle to generate strain curves. These curves represent the deformation of different regions of the aortic wall, with higher strain indicating greater wall deformation and potential risk of aneurysm rupture (Figure 2 The study was conducted in accordance with the Declaration of Helsinki and approved by the Institutional Review Board of Mayo Clinic (IRB# 14-004151, date of approval 8 April 2015). Ethical approval was required because ultrasound images of patients were obtained.) [73]. Ultrasound is a valuable imaging tool due to its safety, absence of ionizing radiation, cost-effectiveness, and portability, making it accessible for routine diagnostics and adaptable for various healthcare settings, including remote locations and real-time evaluations (Figure 2) [75].

## 3. Aortic Calcification

The calcification of arterial walls leads to increased stiffness and rigidity, compromising the structural integrity of the abdominal aorta. This calcification reduces the vessel’s elasticity and causes stress concentrations that may weaken the aneurysm wall [75,76,77,78]. Although the thicker, calcified wall may compensate for its diminished strength by maintaining a failure tension (strength × thickness) similar to that of a thinner, non-calcified wall, studies by Li et al. indicate that calcification actually increases peak wall stress in AAA [79,80]. This suggests that calcification decreases the biomechanical stability of the aneurysm, elevating the risk of rupture. Additionally, experimental studies by O’Leary et al. further highlight that the junction between calcified deposits and the surrounding fibrous matrix is particularly vulnerable to failure, heightening rupture risk [81]. Recent findings by Mansouri et al. reveal that both the number of calcifications and the Euclidean distance between them correlate with an elevated risk of rupture [82].

Calcium scoring is a non-invasive method used to quantify the extent of calcification in the arteries, typically performed using CT scans. The Agatston method is commonly employed, which multiplies the area of calcified plaques by a density factor to provide a calcium score [83,84]. Calcified areas with densities above 130 Hounsfield units (HU) are identified and the area is multiplied by a weighted factor based on the HU range. The final score is the sum of these weighted areas across different segments of the abdominal aorta and iliac arteries, with higher scores indicating a greater calcification burden [85]. Calcium scoring is associated with cardiovascular health, where high calcium scores are linked to increased predictions of mortality and morbidity [86]. By measuring the calcification burden, calcium scoring provides clinicians with a valuable insight into a patient’s overall surgical risk and AAA stability and progression, thus guiding more informed decisions on intervention and treatment.

## 4. Artificial Intelligence

The rapid advancement of AI has significantly broadened the scope for personalizing AAA management. AI enables the integration of multiple indices, such as patient demographic and medical history data, calcium scores, wall stress, and geometric data, providing a comprehensive assessment of a patient’s surgical risk and propensity for rupture. This allows for more precise, individualized evaluations of AAA and supports decision-making on interventions tailored to each patient’s unique risk profile [87].

AI also plays a crucial role in accounting for confounders, which can obscure true relationships between variables in observational studies [88]. Traditional methods like propensity score matching and inverse probability weighting have been used to adjust for confounders, but AI improves upon these approaches by allowing for more accurate data modeling [88]. Machine learning, for instance, is increasingly used to estimate confounders, offering a superior bias-reduction compared to standard regression models. Moreover, AI automates the confounder adjustment process, particularly in large datasets, enhancing the precision of causal inferences [88,89]. This capability is especially valuable in AAA research, where factors like ILT, calcification, and wall stress significantly impact aneurysm progression and rupture risk [90,91,92].

Several studies have demonstrated the value of AI in enhancing AAA risk prediction and image analysis. AI can automate time-consuming processes such as image segmentation and wall stress prediction, improving efficiency and accuracy. Chung et al. developed an AI algorithm that reconstructs three-dimensional (3D) geometries in just 20 s, a reduction from the four hours needed for manual processing while maintaining quality [93]. Similarly, Raffort et al. reviewed 34 studies that utilized AI to improve image segmentation and quantitative analysis of AAA morphology, geometry, and fluid dynamics, showcasing AI’s ability to enhance the efficiency and precision of traditional imaging techniques [90].

AI has also advanced risk prediction beyond traditional measures. Liljeqvist et al. demonstrated that machine learning models incorporating geometric and biomechanical data outperformed traditional clinical assessments based solely on aneurysm diameter. Their model improved the prediction of AAA reaching surgical thresholds and their growth rates [91]. Additionally, Forneris et al. highlighted the use of AI in predicting AAA rupture risk by integrating multiple factors such as time-averaged wall shear stress (TAWSS), ILT, and strain. Using the Extra Trees Classifier, their model achieved a high area under the receiver operating characteristic curve (AUC) of 0.92, indicating the ability of AI to assess local diametric growth accurately [94].

Moreover, AI can predict patient outcomes based on imaging data. Chung et al. developed a machine learning model that predicted AAA outcomes, such as stability, the need for intervention, or rupture, with high accuracy—achieving AUCs of 0.90 for stable aneurysms, 0.80 for repairs, and 0.91 for ruptures [95]. This demonstrates AI’s potential to significantly enhance clinical decision-making and outcome prediction, driving more personalized patient care.

## 5. Contemporary Endovascular Technology

Endovascular aneurysm repair (EVAR) remains the preferred treatment modality for most patients with anatomically suitable AAA. However, for patients with challenging aortic anatomy and advanced pathology, standard endografts are not recommended, as per current guidelines. In such cases, alternative solutions are advised to ensure effective AAA exclusion and long-term success of the repair. Custom-manufactured branched and fenestrated EVAR devices (B/FEVAR) can be utilized for complex anatomies, yet their use is often limited by long manufacturing times (4 to 6 weeks), stringent anatomical requirements, and high procedural costs. Physician-modified endografts (PMEGs) have emerged as a technique to overcome some of the limitations posed by custom-made B/FEVAR devices [96,97]. PMEGs provide more flexibility by allowing intraoperative modifications tailored to the patient’s anatomy. The treatment of descending thoracic aortic pathologies has largely shifted towards endovascular approaches, with a particular focus on managing the challenges of spinal cord ischemia and ensuring adequate perfusion to the left subclavian artery. Advances in endovascular technology, including PMEG and custom-made devices, have expanded treatment options, enhancing safety and effectiveness. In contrast, the aortic arch remains a significant challenge due to its complex anatomy. The curvature of the arch introduces unique flow dynamics and pressure-related forces that complicate endovascular procedures. Furthermore, interventions must preserve the integrity and perfusion of the supraaortic vessels, which are critical for cerebral blood flow. Despite recent advancements, including branched and fenestrated stent grafts, achieving optimal outcomes in the aortic arch requires meticulous planning and specialized techniques due to these anatomical and hemodynamic complexities [98].

Current endografts are constructed from materials that are stiffer than the native aorta [99]. As a result, EVAR is associated with increased vascular stiffness, which has been associated with left ventricular (LV) hypertrophy and diastolic dysfunction [100]. With advancements in understanding arterial wall biomechanics and fluid dynamics, the development of unconventional endografts has been pursued to address these limitations. One such approach is the Multilayer Flow Modulator (MFM) stent, a self-expanding, three-dimensional wire mesh with multiple interconnected layers designed to address the mechanical shortcomings of traditional aortic endografts. The MFM stent permits blood flow through its mesh while modulating laminar flow within the device and aneurysm sac. This flow modulation supports the formation of new endothelial tissue over the aneurysm ostium, isolating the aneurysm from circulation while maintaining aortic compliance [101,102]. However, despite its innovative design, the MFM stent ultimately proved ineffective at limiting aneurysm sac expansion and thus fell out of favor [103]. Nonetheless, the concept of developing devices that better align with aortic wall biomechanics and fluid dynamics remains promising.

Given these limitations, there is a growing need to shift focus towards addressing the underlying pathology rather than attempting to adjust the anatomy to accommodate endografts. Evolving techniques and innovative approaches that treat the aneurysm pathology itself, rather than relying on anatomical modifications, could provide more durable and effective solutions for managing complex AAA cases.

## 6. Emerging Treatments

AAA progression unfolds in three distinct stages. Initially, the degradation of elastic lamellae in the tunica media weakens the vessel and triggers inflammation. In the second stage, small AAAs experience a temporary recovery of collagen; however, this collagen is disorganized and lacks proper elasticity, leading to further vessel expansion. In the final stage, a significant loss of smooth muscle cells, increased thrombosis, and reduced collagen synthesis cause further weakening of the aortic wall, greatly elevating the risk of rupture.

Currently, there is no effective therapeutic treatment available to prevent, delay, or reverse AAA progression beyond reducing modifiable risk factors such as smoking and blood pressure and managing lipid levels. Research has been focused on identifying genomic and epigenetic regulators of AAA pathogenesis with the aim of discovering novel therapeutic targets. Matrix metalloproteinases (MMPs) and their inhibitors play a key role in remodeling the aortic vessel wall. Elevated MMP activity in aneurysmal tissue leads to the degradation of key structural components like elastin and collagen, while a reduction in inhibitors, such as tissue inhibitors of metalloproteinases (TIMPs), further disrupts this balance, promoting the aneurysm’s progression [104]. Wilson et al. reported a localized increase in MMP-8 and MMP-9 at the site of AAA rupture based on their pathological assessment [105]. Moreover, Abdul-Hussien et al. analyzed samples from both ruptured and non-ruptured AAAs and observed that ruptured AAAs were associated with an increase in type I collagen carboxyterminal telopeptide fragments [106]. However, no specific circulating biomarker concentrations have been conclusively linked to AAA rupture [107].

Several pharmacological treatments have been investigated. Doxycycline, an MMP inhibitor, has been studied extensively but has yielded limited success in clinical trials. Other treatments, such as antihypertensives, antiplatelet agents like ticagrelor, immunomodulators such as cyclosporin A, and metformin—which targets inflammation and vascular smooth muscle cell (VSMC) function—have also been explored. However, none have shown definitive effectiveness in halting aneurysm growth [108]. Singh et al., in their Telmisartan in the Management of Abdominal Aortic Aneurysm (TEDY) trial, reported that telmisartan administration was associated with a slower increase in PWS and peak wall rupture index (PWRI) compared to a placebo. However, after adjusting for systolic blood pressure (SBP) at one year, no significant difference between the two groups was observed [109]. Moreover, the Fenofibrate Therapy on Circulating Markers of Abdominal Aortic Aneurysm (FAME-2) trial demonstrated that administering 145 mg/day of fenofibrate for 24 weeks did not significantly reduce AAA growth rates [110]. Of the promising pharmacologic targets, the inhibition of prostaglandin E2 (PGE2) formation via microsomal prostaglandin E synthase-1 (mPGES-1) inhibitors shows potential for slowing AAA progression [108].

Emerging therapeutic strategies also include stem cell therapy, which holds promise to not only inhibit the degradation of the aortic wall but also regenerate the elastic matrix. Stem cells can differentiate into smooth muscle-like cells, contributing to the production of elastin and repairing the damaged aortic wall. In addition, they have the capacity to decrease MMP activity, thereby slowing the breakdown of the matrix and potentially halting aneurysm progression [111]. This dual mechanism of promoting matrix regeneration and preventing further degradation makes stem cell therapy an exciting, nonsurgical treatment option for AAA [112,113]. Mesenchymal stem cells (MSCs) have shown promise in reducing aneurysm size, enhancing elastin in the aortic wall, and decreasing inflammatory markers (e.g., monocyte chemoattractant protein-1, tumor necrosis factor-alpha, interleukin-6) when delivered locally, owing to their immunomodulatory effects [114]. Additionally, biological molecules like growth factors, signaling molecules, and microRNAs (e.g., miR-21 and miR-133a) are being explored for their potential to stabilize aneurysms by influencing cell proliferation, apoptosis, and fibroblast function in vascular smooth muscle cells, offering new avenues for standalone or adjunct therapies [115,116].

**Figure 3:**
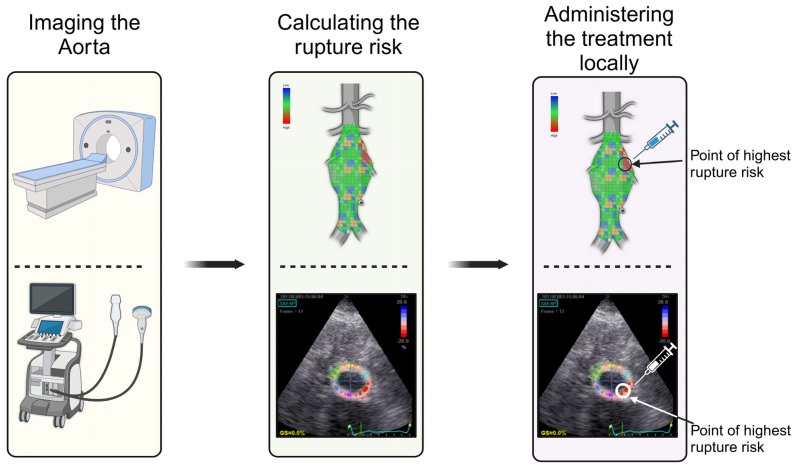
Personalizing treatment for each patient begins with assessing the biomechanics of the abdominal aortic aneurysm using imaging modalities such as computed tomography (CT) and ultrasound (as depicted here). The region of interest with the highest rupture risk is identified. This area is then treated with locally administered therapy.

Another promising avenue in AAA treatment is the use of nanoparticles for enhanced drug delivery. Nanoparticles offer precision targeting, allowing therapeutic agents to be delivered directly to the aneurysm site [117]. They have the advantage of prolonged circulation times and reduced side effects. Nanoparticles can be used to deliver various drugs, including MMP inhibitors and anti-inflammatory agents such as rapamycin and statins [117]. By improving the precision and efficacy of drug delivery, nanoparticle-based therapies could revolutionize the way AAAs are treated, reducing the need for surgical intervention. CRISPR-Cas9 (clustered regularly interspaced short palindromic repeats-associated protein 9) technology has revolutionized genome editing by enabling precise gene modifications through double-strand DNA breaks, repaired by nonhomologous end joining or homology-directed repair. Advanced CRISPR systems facilitate gene regulation and more efficient genetic modifications. High-throughput CRISPR screens, combined with single-cell technologies like Perturb-seq, allow comprehensive studies of gene function and disease mechanisms, particularly in vascular diseases. Additionally, CRISPR enhances disease modeling using iPSC-derived cells and animal models, providing insights into conditions like aortic aneurysms and paving the way for precision therapies [118,119]. CRISPR-based gene editing represents a novel strategy with the potential to target specific genetic mutations and molecular pathways implicated in AAA, including MMP inhibition or the correction of mutations in collagen and elastin production. Chen et al. identified 173 differentially expressed genes associated with AAA that could serve as potential therapeutic targets [120]. These genes hold promise for targeted interventions, but further research is needed to fully understand their roles and establish their associations with AAA progression. Although it offers a potentially curative approach, challenges such as efficient delivery to aortic tissues and mitigating off-target effects remain to be addressed (Figure 3) [121,122].

## 7. Limitation

Implementing advanced techniques in AAA management faces several challenges, including the complexity and high cost of biomechanical modeling and advanced imaging, which rely on patient-specific data that are difficult to obtain and standardize. The integration of AI is limited by the need for large, unbiased datasets, regulatory hurdles, and challenges in clinical adoption. Emerging therapies such as stem cells, nanoparticles, and CRISPR gene editing are still in early stages, with limited clinical validation and delivery challenges. Additionally, the personalized medicine approach demands robust infrastructure, interdisciplinary collaboration, and sophisticated risk stratification models, all of which pose significant barriers in resource-constrained settings.

## 8. Future Direction

The future management of AAA could benefit from prioritizing the validation of advanced screening modalities that integrate biomechanical parameters to more accurately determine when intervention is warranted. Enhancements in imaging technologies, including ultrasound elastography, CT angiography, and MRI, can enable more detailed assessments of wall stress, wall strength, and hemodynamic factors. Incorporating artificial intelligence and machine learning can further refine rupture risk assessments by analyzing complex datasets and controlling for confounding variables, leading to more personalized and precise evaluations beyond traditional measures like aneurysm diameter. This approach could include integrating key biomarkers such as matrix MMPs and TIMPs, which are associated with AAA rupture. Using less invasive techniques, such as analyzing blood samples to detect these biomarkers, and incorporating these data into AI-driven models can provide a comprehensive and non-invasive method for determining rupture risk and identifying optimal timing for intervention. This paradigm shift allows for more tailored treatment strategies, enabling early repair for patients with small aneurysms who have a high rupture risk, while patients with large aneurysms who have a low rupture risk and significant comorbidities can be managed conservatively with close monitoring.

Additionally, developing targeted treatments to reinforce stressed regions of the aortic wall presents a promising strategy to halt aneurysm progression. Advanced imaging and biomechanical modeling can precisely identify regions of high wall stress, allowing for localized delivery of therapeutics such as nanoparticle-based drugs, stem cell therapy, or gene-editing tools like CRISPR-Cas9. These interventions aim to strengthen the aortic wall at its most vulnerable points, potentially reducing the need for surgical procedures and improving overall patient outcomes.

## 9. Conclusions

The management of AAA is evolving as personalized medicine and AI-driven technologies gain prominence. By integrating biomechanical parameters, advanced imaging techniques, and innovative therapeutic approaches, clinicians are moving towards more individualized and precise assessments of AAA evolution and associated risks. Emerging treatments, such as stem cell therapy and nanoparticle-based drug delivery, hold promise for altering the course of AAA progression, potentially minimizing the need for invasive interventions. These innovations highlight a new era in AAA care, offering substantial potential to improve patient outcomes and redefine standards in aneurysm management.

## Figures and Tables

**Table 1:** Summary of indices, their definition, and association with rupture risk.

Index	Definition	Rupture Risk
Peak Wall Stress (PWS)	The maximum mechanical stress experienced by the aneurysm wall	↑
Strain-to-Strength Ratio	The ratio of wall strain (deformation) to wall strength (resistance to deformation)	↑
Rupture Potential Index (RPI)	A combined metric of wall stress and wall strength, assessing stress in weakened areas	↑
Finite Element Analysis Rupture Index (FEARI)	A patient-specific stress distribution model using finite element analysis	↑
Wall Shear Stress (WSS)	The frictional force exerted by blood flow against the aneurysm wall	↑
Tortuosity Index	A measure of the degree of twisting or curvature of the aneurysm	↑
Calcification	The deposition of calcium in the arterial wall	Variable
Intrathoracic Luminal Thrombus (ILT)	A blood clot formed within the aneurysm sac	↑
Elasticity	The ability of the aortic wall to stretch and recoil to its original shape	↓

## Data Availability

Not applicable.
